# Efficient foam-based thermal interface material functionalized with MWCNTs for CPU cooling applications: thermal performance modeling and Experimental studies

**DOI:** 10.1038/s41598-026-41260-5

**Published:** 2026-03-28

**Authors:** Nehal Ali, Badawi Anis, Mohamed Elhadary

**Affiliations:** 1 Department of Engineering Physics and Mathematics, Faculty of Engineering, Tanta University, Tanta, Egypt; 2 Faculty of Applied Health Sciences Technology, Nile Valley University Egypt, Fayoum, 63518 Egypt; 3 Spectroscopy Department, Physics Research Institute, National Research Centre, Giza, Cairo, 12622 Egypt; 4 Department of Mechanical Power Engineering, Faculty of Engineering, Tanta University, Tanta, Egypt

**Keywords:** PVF/MWCNT foams, TIM, Modeling, Thermal analysis, Energy science and technology, Engineering, Materials science, Nanoscience and technology, Physics

## Abstract

Efficient thermal dissipation remains one of the foremost challenges in modern electronics, as excessive heat can critically damage device performance and reliability. In this work, we introduce a novel thermal interface material (TIM) based on polyvinyl-formaldehyde (PVF) foam functionalized with multi-walled carbon nanotubes (MWCNTs) for advanced processor cooling. The developed composite exhibits high thermal conductivity, remarkable stability up to 200 °C, and minimal weight loss across a wide temperature range. A TIM with a $4\times 4$ cm^2^ cross-section was engineered to interface directly with a CPU chip. The heat dissipation from the CPU was systematically investigated as a function of composition and design parameters to identify the optimal cooling configuration. Thermal characterization and CPU package modeling confirmed the superior heat dissipation capacity of the TIM. Among the tested configurations, the PVF/MWCNT composite with 4 wt% loading, fabricated in a square geometry and 2 mm thickness, demonstrated the most effective performance, achieving a minimum CPU temperature of 66.72°C under an 80 W heat load. The square-shaped TIM outperformed its circular counterpart due to better conformity with the CPU surface, maximizing contact area and minimizing thermal resistance. Experimental validation closely matched the simulation results, confirming the reliability of the adopted model. These results establish PVF/MWCNT composites as a lightweight, thermally stable, and highly efficient TIM, offering strong potential for next-generation electronic devices operating at elevated temperatures.

## Introduction

Excessive heat generation from high power densities and continuous operation critically undermines the efficiency, operational lifespan, and reliability of electronic components^1,2^. The global drive toward device miniaturization and multi-functionality leads to sharply increased heat flux, elevating the risk of thermal failure^3,4^. Effective thermal management has therefore become a primary bottleneck for technological advancement, particularly in applications like CPUs and LCDs where localized hot spots can throttle performance and accelerate failure^5,6^.

A key element in thermal management is the thermal interface material (TIM), which bridges the semiconductor die and the heat sink by filling microscopic air gaps, thereby minimizing interfacial thermal resistance^7,8^. While conventional cooling methods like fans suffice for standard chips, they are inadequate for high heat-flux microchips, creating a pressing need for advanced TIMs with superior thermal conductivity and stability^9–11^.

Multi-walled carbon nanotubes (MWCNTs) are highly promising for this role due to their exceptional intrinsic axial thermal conductivity (up to ~ 3000 W/m·K)^12–14^. The primary challenge, however, lies not in the nanotubes themselves but in effectively integrating them into a practical, functional TIM that overcomes issues like aggregation, poor mechanical stability, and difficult processing^15,16^. Early approaches using vertically-aligned MWCNT arrays showed potential but faced limitations such as high synthesis temperatures and poor interfacial compliance^17^.

A promising strategy to address these issues is embedding MWCNTs into polymeric foams. This approach combines the mechanical support, flexibility, and low cost of the polymer matrix with the enhanced thermal pathways provided by the nanotubes^18–22^. The porous foam structure improves conformability to rough surfaces and facilitates large-area integration, which is essential for practical microelectronics cooling.

Recent work on MWCNT/polymer composites has demonstrated thermal conductivities typically in the range of 2–10 W/m·K, influenced by CNT loading, alignment, and dispersion quality^23–28^. Foam-based systems, in particular, have shown enhanced potential due to their low density and ease of fabrication. For instance, MWCNT-doped polyvinyl-formaldehyde (PVF) foams with ~ 4 wt% loading have achieved thermal conductivities approaching 15 W/m·K, highlighting their viability as lightweight, cost-effective TIMs^29,30^.

Building on this foundation, this study investigates PVF foams functionalized with MWCNTs as a novel TIM system. By leveraging the synergistic combination of nanotube thermal transport and the structural advantages of polymer foams, the proposed TIM is designed to deliver high thermal conductivity, mechanical robustness, and stable performance at elevated temperatures. The work employs experimental characterization and CPU package simulations to evaluate thermal dissipation performance, with a specific focus on the effects of composition, thickness, and structural design.

## Materials and methods

### Modeling parameters

ANSYS Icepak is a CAE software that allows the modeling of heat transfer simulations based on the finite volume method using a SIMPLE algorithm. For the selected geometries, the flow is considered turbulent based on Reynolds averages of the governing equations. The solution variables in the instantaneous Navier–Stokes equation are decomposed into the mean and the fluctuating components. The governing equations for the fluid and solid regions are solved using ICEPAK. R18. The segregated solver is the solution algorithm used in this study, which solves the governing equations of mass, momentum, and energy sequentially.

#### Assumptions of the model

The simulations were conducted under a set of simplifying assumptions to depict the computational model tractable while maintaining physical relevance. Steady-state heat transfer was assumed, as transient effects were not the primary focus of this study. The thermal interface material was treated as homogeneous and isotropic, with uniform distribution of MWCNTs within the foam matrix. Radiation heat transfer was considered negligible compared to conduction and convection. For the simulation, perfect thermal contact was assumed at all material interfaces (e.g., CPU-TIM and TIM-heatsink). Ambient temperature and the performance characteristics of the cooling fan were held constant throughout the analysis.

### Statement of the problem and method of solution

Active CPU coolers are one of the effective technologies to remove heat from highly integrated CPUs. In this work, an active cooling unit consists of an aluminum radial heat sink and an innovative thermal interface material made of PVF/MWCNTs are designed to cool a CPU and dissipate 80W of heat. This value of dissipated watts considered quite typical and can be considered normal for many modern processors, especially high-performance desktop or server CPUs.

**Fig. 1:**
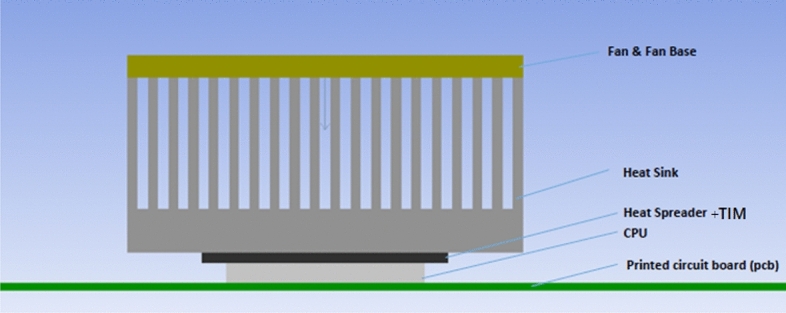
Schematic of the Facade view of the suggested model of the active CPU cooler.

The active CPU cooler shown in Fig. 1 is composed of an axial fan sitting above the heat sink, an aluminum radial heat sink, and the TIM at the base of the heat sink. CFD (Computational Fluid Dynamics) simulations have been used to explore the effect of functionalized foam material as a TIM and heat sink of the processor. A parametric study was also conducted to determine the maximum heat this design can dissipate to cool the CPU effectively.

The model considers the following parameters: The fan (model delta FFB-0812-24EHE) is an axial fan with dimensions 72 mm × 72 mm with extruded aluminum fins of 30 cross-cut extrusion and 0.07 m in height. The maximum fan speed is 5700 rpm. The fan is placed above the heat sink and is attached to a PCB board, which houses a CPU package with a surface area of 40 mm × 40mm and 80 W of heat generation. We carried out the tests in three stages. At first, we used the foam composite as a TIM with different percentages of MWCNT, which in turn have different thermal conductivities, as shown in Table 1. Second, the investigation concentrates on the optimum thickness of the best result of the first stage. In the third investigation, we focus on the shape of the TIM with the best thickness from the second stage.

**Table 1:** Thermal conductivities of the corresponding case notation for each percentage of MWCNT^29^.

	Case 1	Case 2	Case 3	Case 4
Wt % of MWCNT	0	1	2	4
λ (W/m.K)	0.035	10	12	15

### Governing equations

The three-dimensional steady-state, turbulent compressible flow in the cabinet is governed by continuity, momentum, and energy equations together. The governing equations of the flow are modified according to the conditions of the simulated case. Since the problem is assumed to be a steady state with low velocities. Therefore, time-dependent parameters are dropped together with the viscous dissipation term from the equations. For steady-state, three-dimensional turbulent flow, the governing equations in Cartesian coordinates are written as follows are^31^:

*Continuity equation*

$$\frac{\partial \left(\rho u\right)}{\partial x}+\frac{\partial \left(\rho v\right)}{\partial y}+\frac{\partial \left(\rho w\right)}{\partial z}=0$$

*Momentum equations*

- *x-momentum*
  $$\rho \left(u\frac{\partial u}{\partial x}+v\frac{\partial u}{\partial y}+w\frac{\partial u}{\partial z}\right)=-\frac{\partial p}{\partial x}+\mu \left(\frac{{\partial }^{2}u}{\partial {x}^{2}}+\frac{{\partial }^{2}u}{\partial {y}^{2}}+\frac{{\partial }^{2}u}{\partial {z}^{2}}\right)$$
- *y-momentum*
  $$\rho \left(u\frac{\partial v}{\partial x}+v\frac{\partial v}{\partial y}+w\frac{\partial v}{\partial z}\right)=-\frac{\partial p}{\partial y}+\mu \left(\frac{{\partial }^{2}v}{\partial {x}^{2}}+\frac{{\partial }^{2}v}{\partial {y}^{2}}+\frac{{\partial }^{2}v}{\partial {z}^{2}}\right)$$
- *z-momentum*
  $$\rho \left(u\frac{\partial w}{\partial x}+v\frac{\partial w}{\partial y}+w\frac{\partial w}{\partial z}\right)=-\frac{\partial p}{\partial z}+\mu \left(\frac{{\partial }^{2}w}{\partial {x}^{2}}+\frac{{\partial }^{2}w}{\partial {y}^{2}}+\frac{{\partial }^{2}w}{\partial {z}^{2}}\right)$$

*Energy equation*

$$\rho {c}_{p}\left(u\frac{\partial T}{\partial x}+v\frac{\partial T}{\partial y}+w\frac{\partial T}{\partial z}\right)=k\left(\frac{{\partial }^{2}T}{\partial {x}^{2}}+\frac{{\partial }^{2}T}{\partial {y}^{2}}+\frac{{\partial }^{2}T}{\partial {z}^{2}}\right)+q$$

where $u,v,w$ are velocity components in the $x,y,z$ directions, $\rho$ is the density, $\mu$ is the dynamic viscosity, $k$ is the thermal conductivity, ${c}_{p}$ is the specific heat, and $q$ is the volumetric heat generation term.

Turbulent Modeling The default turbulence model of all calculations is Algebraic Turbulence Model. It is a two-equation model and computationally least expensive since no extra equations are solved in addition to continuity, momentum, and energy equations. However, to rely on the results that the algebraic model gives, it should be validated with higher-order turbulence models. K-ε model was used as a test case. The temperature distributions and velocity fields are compared. The results show acceptable agreement. Therefore, the Algebraic Turbulence Model is suitable for use.

### Model validation experiments

**Fig. 2:**
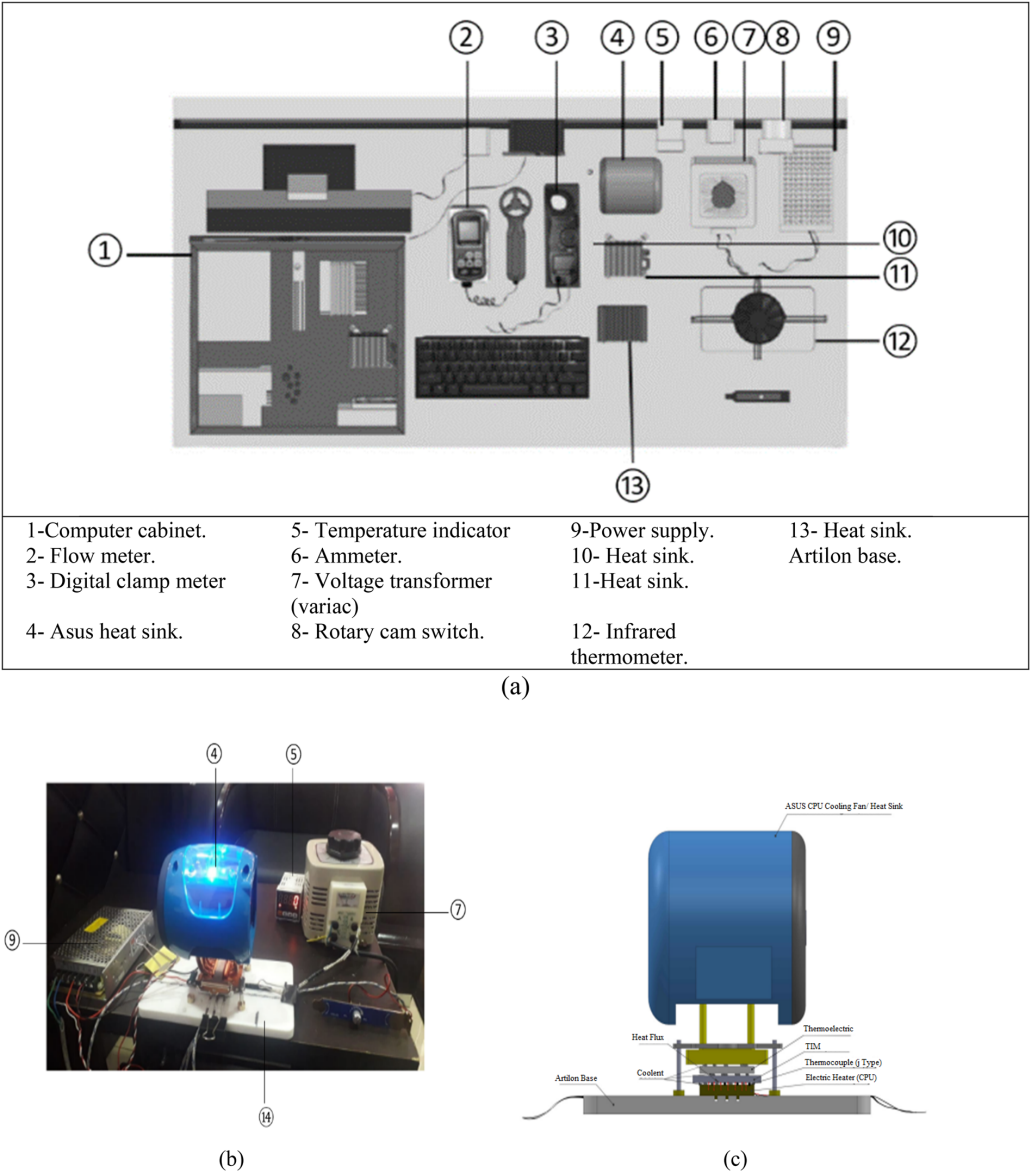
Configuration of the testing: (**a**) Overall test-rig; (**b**) photograph of assembling; (**c**) structure of the heating block. Test–rig of the experimental validation mainly consists of the following components.

The experimental setup is schematically illustrated in Fig. 2a, with a corresponding photographic view shown in Fig. 2b, c. The facility is based on a computer chassis (1), model HP Compaq dc5850, containing a CPU (AMD Phenom HD, model no. 86OBWCJ3BGH). Cooling air is supplied from the ambient environment through a fan (FOXCONN model PV902512PSPFOD), which directs airflow inside the cabinet via a guide, crossing the heat sink (4) (model HP P/N: 450666-001).

The primary air mass flow rate is automatically regulated by a sensor embedded in the motherboard, in response to the heat flux generated by the CPU.

To simulate and control CPU heating, a heater was mounted on an Artilon base (14), producing a heat flux of 5 W/cm^2^. The thermal load was applied through a flat chip (40 × 40 mm^2^) that generated a maximum thermal power of approximately 80 W. The power input to the heater was controlled using a variable voltage autotransformer (7), with current monitored via an ammeter (6). For cooling, heat sinks were fixed above the CPU in combination with a thermoelectric cooler (TEC) (model TEC1-12706). J-type thermocouples (probe size: 1 × 150 mm) were attached directly to the CPU surface to record average temperatures^32^.

The uncertainty of the temperature measurements was estimated at ± 0.5 °C. Air mass flow rate was measured using a flow meter (2) (model TENSE-DFR-72), while temperature readings were displayed using a digital temperature indicator (5) (model Autonics-TC4S-24R). In a preliminary experiment, we increased the heater’s input voltage and recorded the corresponding output temperature to evaluate three cooling setups: without a heatsink, with a heatsink, and with a heatsink combined with a thermoelectric cooler (TEC).

**Fig. 3:**
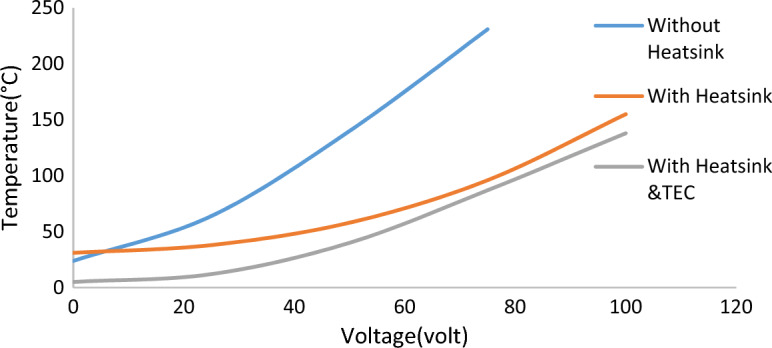
The relation between temperature values at different volt values in three configurations; without Heatsink, with Heatsink, and with Heatsink &TEC.

The curves in Fig. 3 show the temperature response at different voltages for each configuration. As illustrated, the most effective cooling is achieved with the heatsink and TEC combination. However, even with this setup, direct attachment of the cooling element to the processor surface reduced the CPU temperature to a minimum of 71.3 °C, indicating that optimal heat dissipation was still not achieved^33^.

However, when a copper heat spreader of 2mm thickness was inserted on the surface of the cooling element, a significant improvement was observed in the heat distribution, and the max temperature of the CPU was reduced to 69.06˚C. In this work, we will test very cheap and easily fabricated polyvinyl-formaldehyde (PVF) foam functionalized with multi-walled carbon nanotubes (MWCNTs) as TIM and study its thermal distribution to prove their effectiveness in the cooling process of the CPU units.

The experimental setup, while providing controlled conditions for TIM validation, is subject to several practical limitations. Imperfect surface conformity and microscopic air gaps between the CPU, TIM, and heatsink introduce thermal contact resistance.

Although sonication was used to promote dispersion, slight agglomeration of MWCNTs within the PVF foam may lead to local variations in thermal conductivity. Moreover, Temperature measurements using J-type thermocouples carry an estimated uncertainty of ± 0.5 °C, affecting measurement precision. Finally, the experiments were performed in a stable laboratory environment and did not simulate real-world conditions with variable airflow or device orientation.

### Fabrication of TIM

The thermal interface material (TIM), a 4 wt% MWCNT-doped polyvinyl formal (PVF) foam, was synthesized according to the previous work^29^. The procedure consisted of two main stages:

*MWCNT synthesis and purification:* A bimetallic catalyst with a molar ratio of Mo:Fe:MgO = 1:8:91 was prepared using a combustion synthesis method to disperse iron and molybdenum nanoparticles onto a magnesium oxide support. The MWCNTs were then grown via chemical vapor deposition (CVD) in a horizontal 2.5-inch quartz tube furnace. The catalyst was heated to 700 °C under a hydrogen flow, at which point acetylene gas was introduced as the carbon source for a growth period of 30 min. The resulting product was subsequently purified by refluxing in concentrated hydrochloric acid at 60 °C for 24 h to dissolve the metal catalyst and support. The purified MWCNTs were collected via vacuum filtration, washed with deionized water until neutral pH was achieved, and dried in a vacuum oven at 80 °C overnight.

*Foam/MWCNT composite preparation:* The preparation of the MWCNT-doped PVF foam began by dissolving five grams of polyvinyl alcohol (PVA) in 45 mL of distilled water at 95 °C for 3 h under constant mechanical stirring. Concurrently, 0.2 g of the purified MWCNTs were dispersed in 10 mL of an aqueous solution containing 4 wt% Triton-X-100 surfactant using tip sonication for 1 h while maintained in an ice bath. This homogeneous MWCNT dispersion was then added dropwise to the hot PVA solution under vigorous magnetic stirring, which was continued for an additional hour to ensure a uniform mixture. Following this, 10 mL of formaldehyde was introduced as a cross-linking agent and stirred for 10 min. After cooling to room temperature, 30 mL of sulfuric acid (25 wt% aqueous solution) were added dropwise under vigorous stirring to catalyze the acetalization reaction. The complete mixture was transferred to a sealed container and cured in an oven at 60 °C for 6 h to form the cross-linked PVF foam structure. Finally, the resulting MWCNT@PVF foam was cooled, washed exhaustively with hot deionized water to remove residuals, and dried at 40 °C for 24 h.

## Results and discussion

### Calculated distribution of the dissipated heat through the cooling system

The schematic diagram below illustrates that the study focused on calculating the heat dissipated from the CPU unit as a function of different parameters concerning the used material of the TIM from both aspects of composition and design parameters. The following schematic explains the adopted parameters.
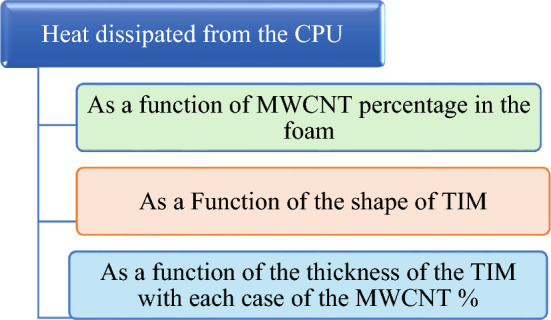


#### The effect of MWCNTs wt% in the composite of the TIM

**Fig. 4:**
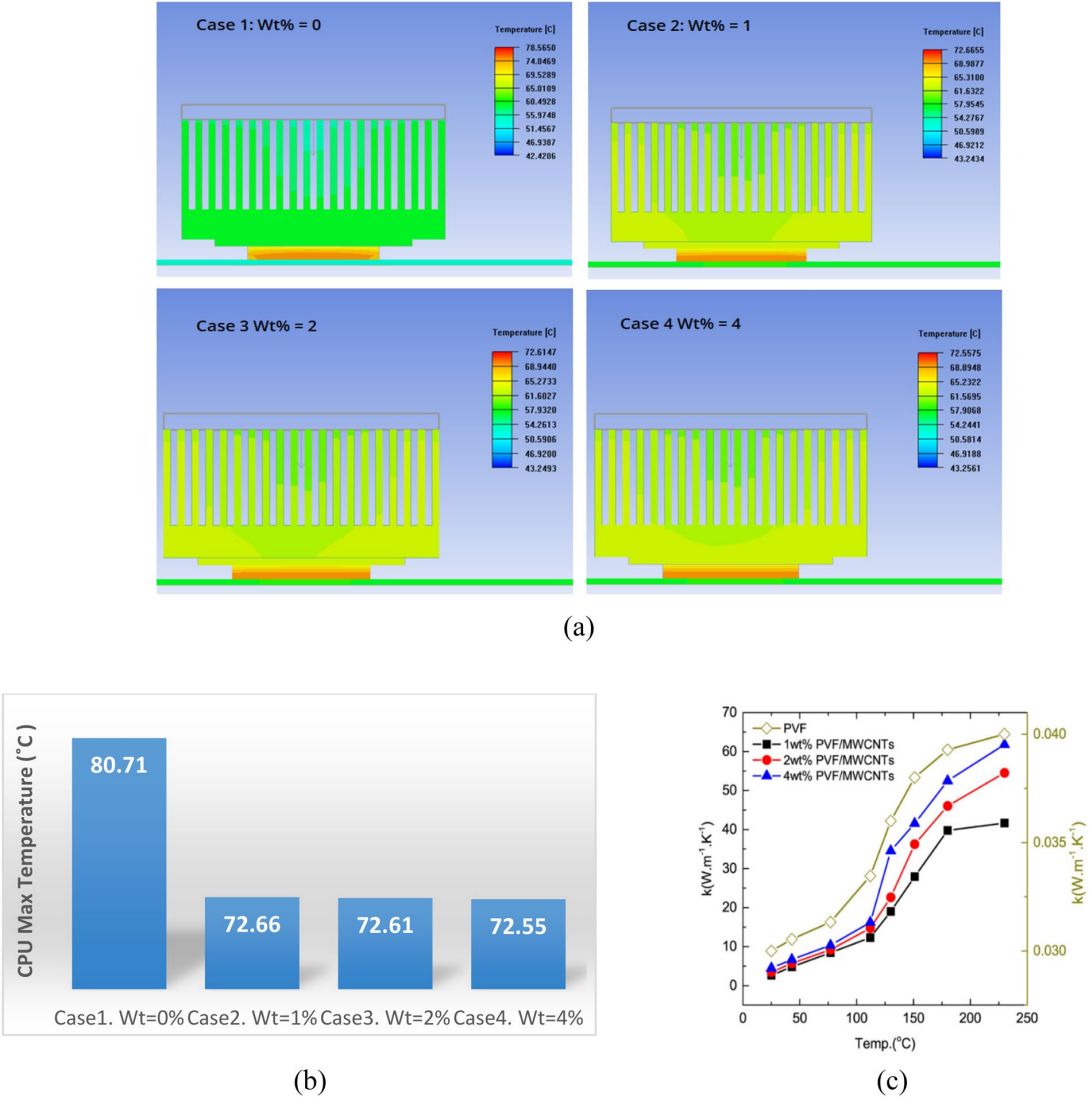
(**a**) Heat distribution through the fan. The effect of wt% of MWCNT/PVF foam (case1. wt = 0%, case2. wt% = 1%, case3. wt   = 2%, case4. wt% = 4%) in the TIM. (**b**) Maximum temperature of the CPU unit curve for the four cases. (**c**) Thermal conductivity for PVF, 1 wt%, 2 wt%, and 4 wt% PVF/MWCNTs foam at temperatures up to 200C. With increasing the MWCNTs content the thermal conductivity increases^29^.

Figure 4a presents the heat dissipation distribution through the cooling system as a function of MWCNTs content in the polyvinyl-formaldehyde (PVF) foam within the TIM. The foam without MWCNT additives exhibits poor heat dissipation through the heatsink, as the fan maintains a nearly constant temperature. By increasing the MWCNT content significantly improves heat distribution, with the most pronounced effect observed in Case 4, where the foam contains 4 wt% MWCNTs.

In this case, the dissipated heat is distributed more uniformly across the fan. As shown in Fig. 4b, the lowest CPU temperature of 72.5 °C is achieved with 4 wt% MWCNTs, demonstrating a substantial enhancement in the cooling system performance due to the increased MWCNT content in the foam. The observed improvement in heat dissipation with the addition of MWCNTs can be attributed to their exceptional thermal conductivity, which is several orders of magnitude higher than that of the polymeric PVF foam. When MWCNTs are incorporated into the foam, they form conductive pathways that facilitate efficient phonon and electron transport, thereby lowering the overall thermal resistance of the TIM^34,35^. At higher loadings (e.g., 4 wt%), these conductive networks become more continuous, allowing heat to spread more uniformly across the foam and into the heatsink. This explains why the heat distribution through the fan becomes more homogeneous and why the CPU achieves a lower steady-state temperature Fig. 4b. In contrast, the pure PVF foam lacks these conductive pathways, resulting in localized hot spots and poor overall heat dissipation.

As established in our group’s previous work^29^, all measurements and analyses of thermal conductivity for PVF/MWCNT foam were carried out and published as illustrated in Fig. 4c. That study reported a maximum thermal conductivity of ~ 15 W/mK at room temperature for the 4 wt% PVF/MWCNT foam which is 166 times higher than that of neat PVF foam and exceeding many previously reported carbon composite heat sinks. Furthermore, the conductivity of the same composition increased to 65 W/mK at 200 °C, as illustrated in Fig. 4c.

#### Effect of the shape of the TIM

**Fig. 5:**
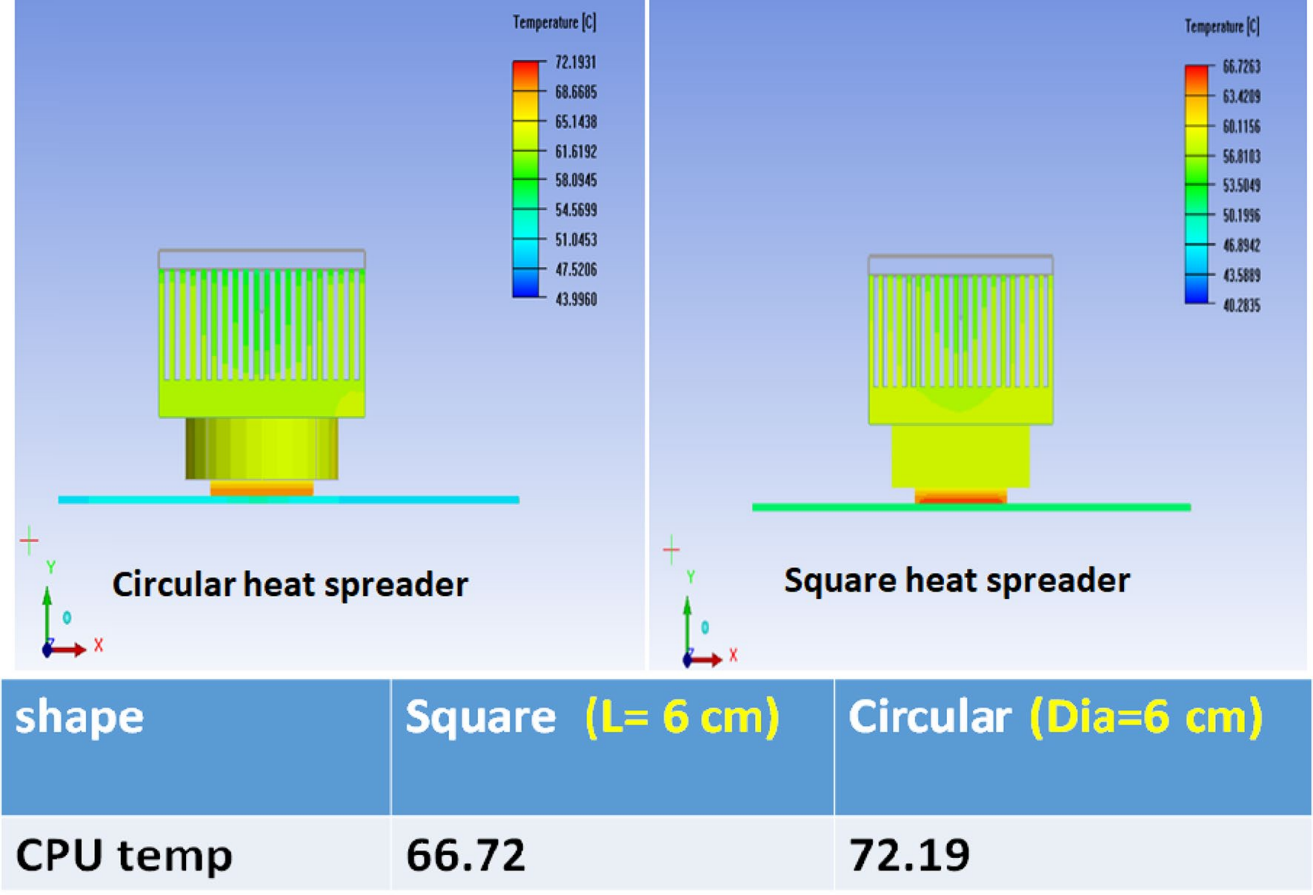
The dissipated heat distribution from the CPU is a function of the shape and dimension of the foam composite. The table shows the optimum CPU temperatures when using circular and square TIM.

The adopted simulation model predicts the CPU temperature for different shapes and dimensions of the TIM. Figure 5 illustrates the heat dissipation distribution from the CPU as a function of the foam composite’s geometry. As summarized in the table, the CPU reached an optimum temperature of 72.19 °C with a circular TIM, compared to 66.72 °C with a square TIM. These results indicate that the square-shaped TIM provides superior cooling performance. The superior performance of the square TIM compared to the circular one can be attributed to its better conformity with the CPU surface geometry. Most CPU chips and their contact areas are manufactured in square or rectangular shapes, meaning that square TIM maximizes the actual contact area and minimizes unused regions at the edges. This reduces interfacial thermal resistance according to the relation ${R}_{c}\propto 1/A$, where ${R}_{c}$ is the contact resistance and $A$ is the actual contact area. Which in turn ensure more efficient heat transfer pathways across the entire CPU–TIM–heatsink interface.

#### Effect of TIM thickness

**Fig. 6:**
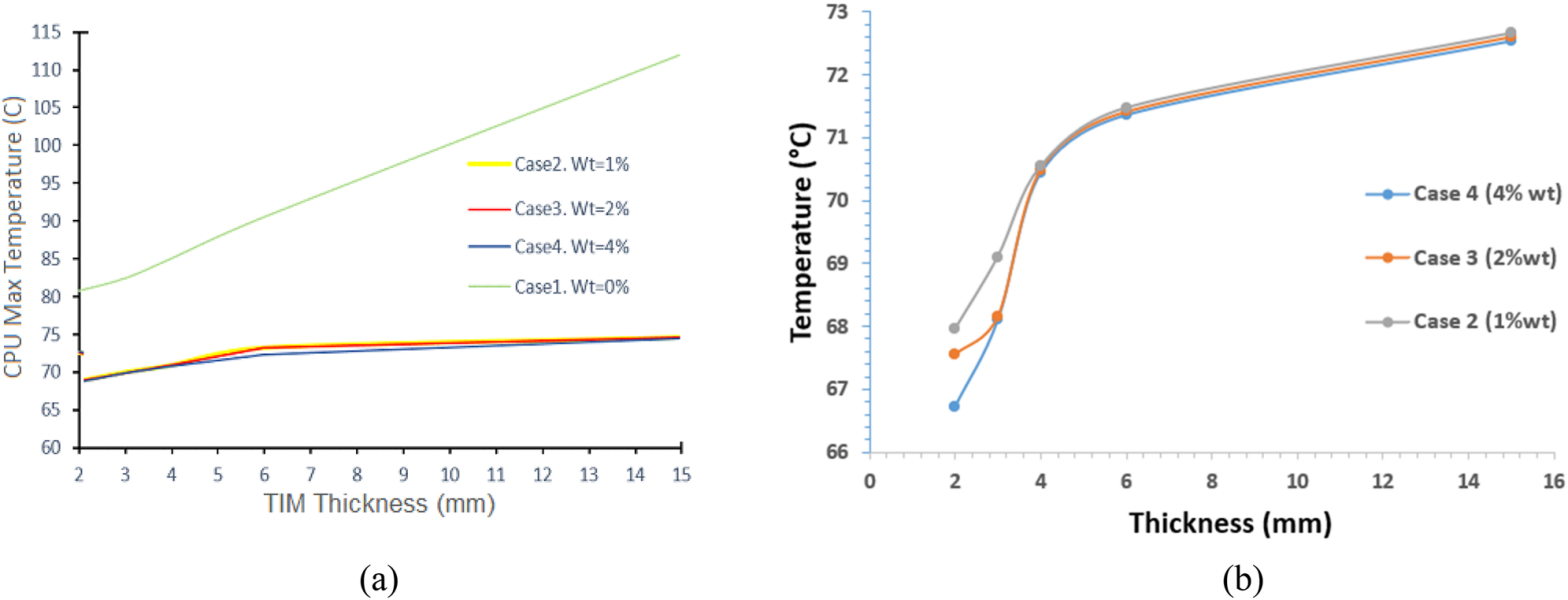
(**a**) the CPU temperatures versus TIM thicknesses in mm, with different percentages of MWCNTs/PVF composite, (**b**) magnification Figure of the curves in Figure (a) for the three cases 2, 3 and 4.

The heat dissipation efficiency of PVF/MWCNTs foam was examined as a function of composite thickness. Thicknesses of 2, 3, 4, 6, and 15 mm were selected to cover a wide practical range, and the results are summarized in Table 2. For the neat foam (Case 1, 0 wt%), the dissipation performance is shown in Fig. 6a. At 15 mm thickness, the CPU temperature reached 112.12 °C, while the lowest temperature recorded for this case was 80.71 °C at 2 mm thickness. Even at its best performance, however, the neat foam remained significantly less effective compared to MWCNT-loaded foams at the same thickness.

**Table 2:** The temperature of the CPU unit as a function of the TIM thicknesses for different compositions of the PVF/MWCNTs foam.

Thickness	Temperature (°C)			
	Case 4 (4% wt)	Case3 (2% wt)	Case2 (1% wt)	Case1 (0% wt)
2 mm	66.72	67.56	67.95	80.71
3 mm	68.10	68.16	69.10	82.39
4 mm	70.44	70.49	70.54	85.03
6 mm	71.37	71.42	71.47	90.53
15 mm	72.55	72.61	72.66	112.12

As shown in the magnified Fig. 6b, the TIMs containing MWCNTs exhibited a consistent trend: decreasing thickness combined with higher MWCNT content enhanced heat dissipation. The most effective configuration was achieved with 4 wt% PVF/MWCNT foam at 2 mm thickness, which yielded the lowest CPU temperature of 66.72 °C. These results highlight the critical role of both filler concentration and optimized thickness in improving TIM performance.

**Fig. 7:**
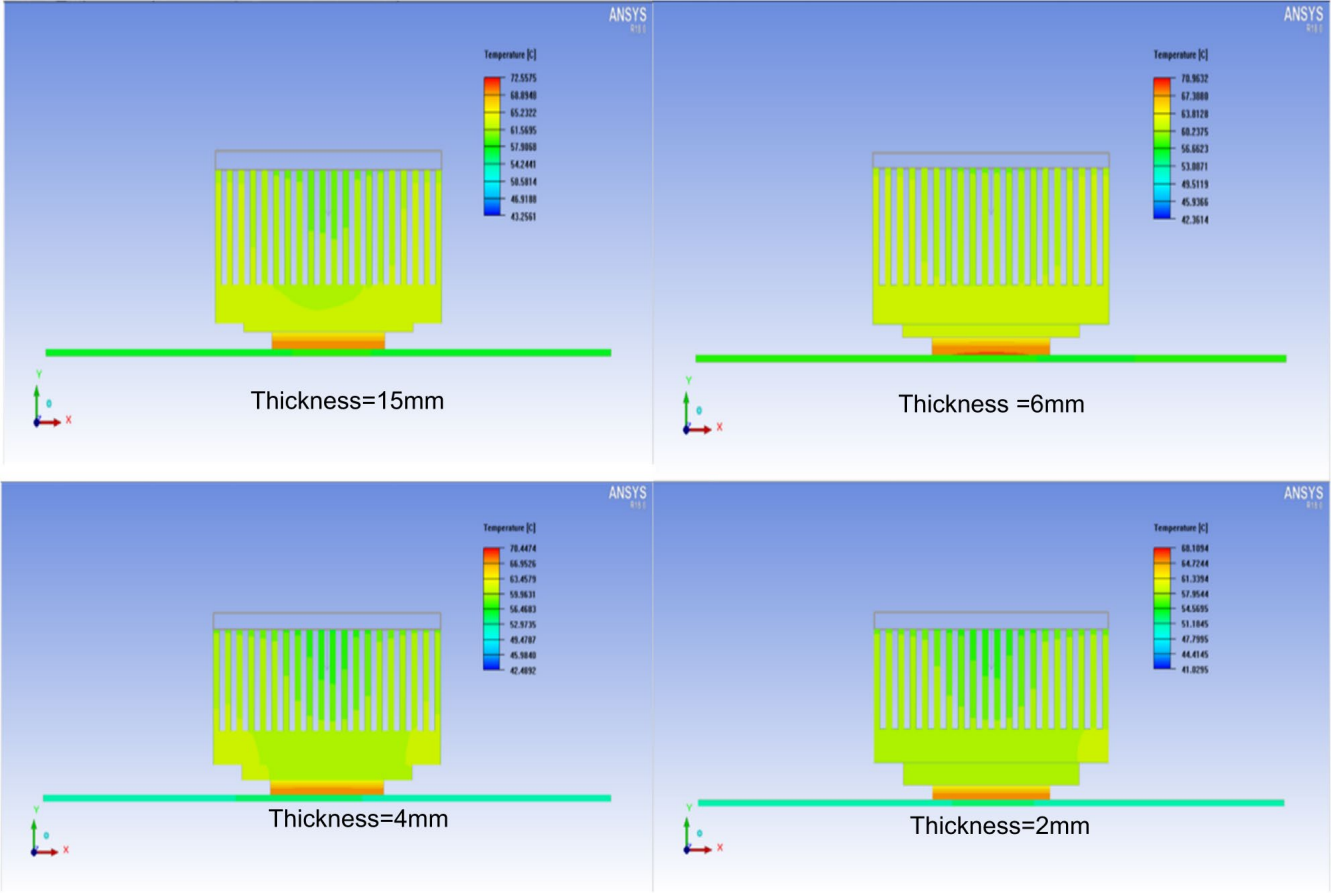
The dissipated heat distribution from the CPU as a function of thickness for case 4 (wt 4%) of MWCNT of the PVF foam heat spreader.

Figure 7 shows the heat dissipation distribution for 2, 4, 6, and 15 mm thicknesses. The results revealed the effect of decreasing thickness on the heat dissipation distribution. The smaller the thickness the better the distribution of the dissipated heat through the cooling fans. As seen in the Figure, the TIM of 4% wt of MWCNTs with a thickness of 2mm gives the optimum performance as a TIM.

The variation in CPU temperature with TIM thickness follows a classical thermal resistance model, where the total resistance ${R}_{tot}$ is given by ${R}_{tot}=\frac{L}{kA}+{R}_{c}$, with $L$ being the TIM thickness, $k$ the effective thermal conductivity, $A$ the area, and ${R}_{c}$ the contact resistance. For a given $k$, increasing $L$ linearly increases the conductive resistance. At 15 mm thickness, even with MWCNTs, the extended conduction path dominates, leading to higher CPU temperatures. At 2 mm thickness, the conductive resistance is minimized, allowing the high intrinsic conductivity of the MWCNT network to dominate heat transfer. Furthermore, thinner TIMs improve conformability under mounting pressure, reducing ${R}_{c}$ and enabling more effective utilization of the filler-enhanced conductivity. The improvement with higher filler loading can be attributed to the formation of more continuous conductive pathways for phonon transport, which further reduces the effective thermal resistance of the composite. This explains why the lowest CPU temperature of 66.72 °C was achieved with 4 wt% MWCNT/PVF foam at 2 mm thickness. These findings are consistent with previous studies showing that thinner TIM layers combined with high-conductivity fillers offer superior performance by balancing bulk conduction and interfacial contact resistance^36–39^.

### Experimental validation results

**Fig. 8:**
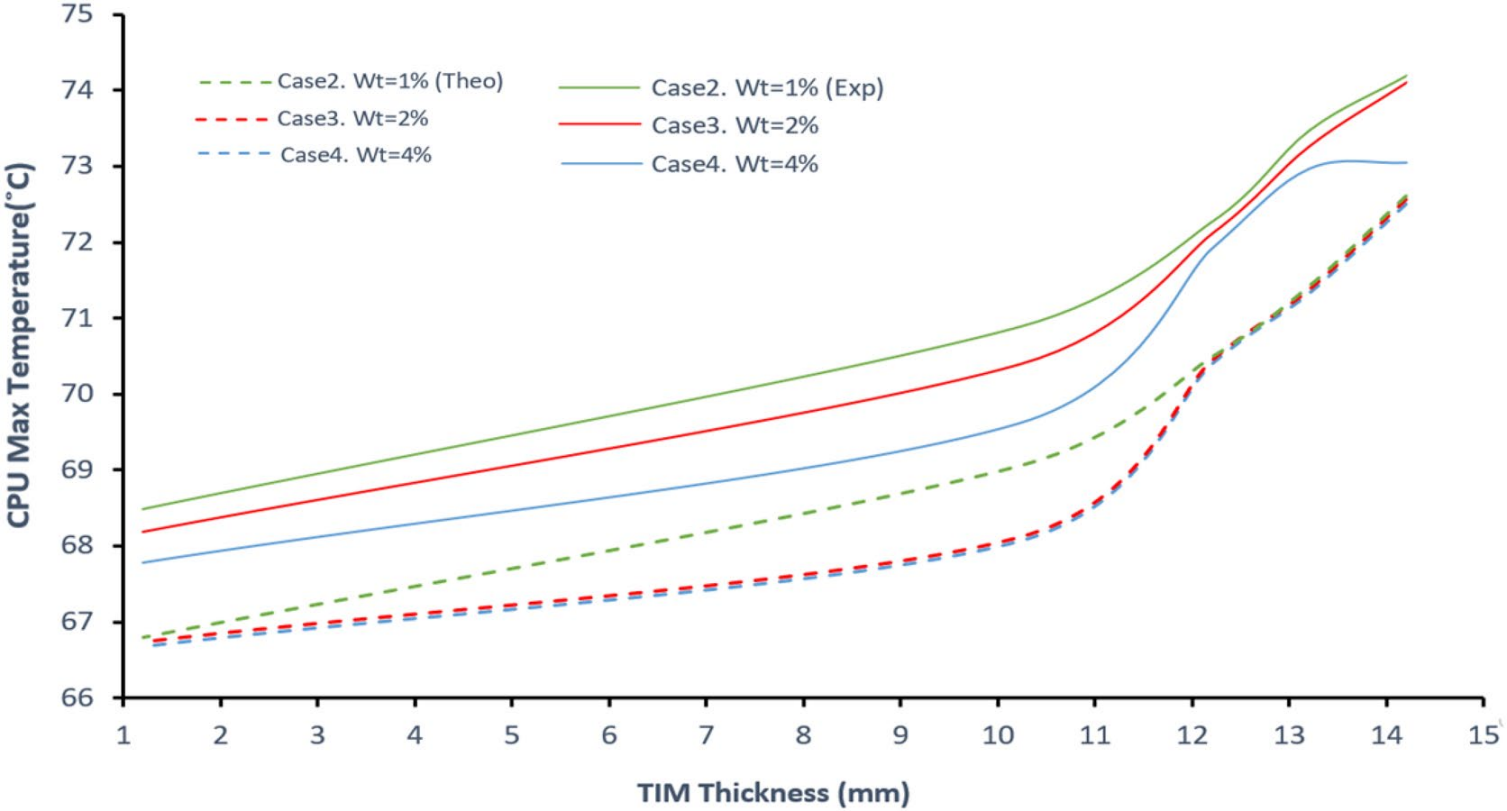
Experimental and theoretical results of the CPU temperature as a function of TIM thickness with different percentages of MWCNTs/PVF, the results of the simulation model (dash lines) with experimental results (solid lines).

Figure 8 presents a comparison between experimental and theoretical predictions of CPU temperature as a function of TIM thickness for varying weight percentages of MWCNTs/PVF composites. The data reveal a clear trend: CPU temperature decreases with both increasing MWCNT content and decreasing TIM thickness. At lower thicknesses (e.g., 2–3 mm), the heat transfer pathway is shorter, leading to reduced interfacial thermal resistance and consequently better cooling performance. The addition of MWCNTs significantly enhances this effect by improving the effective thermal conductivity of the foam matrix, resulting in lower operating CPU temperatures compared to the neat PVF foam.

The close agreement between experimental and simulation results validates the adopted heat transfer model under the tested conditions. However, the minor deviations observed particularly at greater TIM thicknesses can be attributed to the experimental limitations noted in Section "Model validation experiments" Specifically, contact imperfections and filler dispersion heterogeneity become more pronounced as the conduction path lengthens, slightly elevating thermal resistance beyond the idealized model. Furthermore, the fixed heat load and ambient conditions of the experiment, while controlled, do not capture the full range of operational scenarios encountered in field applications. Future work will aim to characterize TIM performance under cyclic thermal loads and in varied mounting configurations to better assess real-world reliability. Overall, the results highlight the synergetic role of filler content and optimized thickness in maximizing TIM performance.

## Conclusion

The continued push toward smaller, more powerful electronic devices is raising heat fluxes to levels that conventional thermal interface materials increasingly struggle to manage. In response to this need, this work developed and evaluated a new foam-based thermal interface material (TIM) for CPU cooling: a polyvinyl-formaldehyde (PVF) foam functionalized with multi-walled carbon nanotubes (MWCNTs). By combining the high axial thermal conductivity of MWCNTs with the low density, compliance, and manufacturability of a polymer foam, the composite creates an efficient and practical heat-transfer bridge between the CPU die and a standard aluminum heatsink.

A coordinated program of computational modeling and experimental testing was used to identify the key levers that control performance. Increasing the nanotube loading to 4 wt% enabled the formation of a percolated conductive network within the PVF matrix, substantially improving effective thermal conductivity and enhancing lateral heat spreading. As a result, peak CPU temperature was markedly reduced relative to the unmodified foam. Geometric optimization further showed that a square TIM footprint outperformed a circular design by better matching the rectangular CPU contact area and reducing interfacial thermal resistance. In addition, thickness optimization indicated that a 2 mm layer of the 4 wt% PVF/MWCNT composite provides the best overall trade-off—minimizing conduction resistance while maintaining sufficient integrity and conformability under mounting pressure—achieving a minimum CPU temperature of 66.72 °C at an 80 W steady-state load.

The close agreement between simulations and experiments supports the validity of the thermal models and confirms the dominant physical mechanisms: percolation-driven conduction combined with contact-area optimization. Beyond its cooling performance, the PVF/MWCNT foam TIM demonstrates thermal stability, a lightweight form factor, and a pathway to cost-effective, scalable manufacturing. These results position PVF/MWCNT foam composites as a strong, practical candidate for next-generation TIMs, offering a credible route to more reliable thermal control in high-performance computing, servers, and increasingly power-dense consumer electronics.

## Data Availability

The data could be available upon request.

## References

[1] Kandlikar, S. G. Review and projections of integrated cooling systems for three-dimensional integrated circuits. *J. Electron. Packag.* **136**, 024001 (2014).

[2] Ersoy, K. Review of electronic cooling and thermal management in space and aerospace applications. *Eng. Proc.* **89**(1), 42 (2025) (**Mar 26;**).

[3] Zhang, H., Sun, F. & Liu, Y. Thermal and mechanical properties of micro Cu doped Sn58Bi solder paste for attaching LED lamps. *J. Mater. Sci. Mater. Electron.* **30**(1), 340–347. 10.1007/s10854-018-0298-0 (2019).

[4] Garimella, S. V., Persoons, T., Weibel, J. A. & Gektin, V. Electronics thermal management in information and communications technologies: Challenges and future directions. *IEEE Trans. Compon. Packag. Manuf. Technol.* **7**, 1191–1205 (2016).

[5] Orville, T., Tajwar, M., Bihani, R., Saha, P. & Hannan, M. A. Enhancing thermal efficiency in power electronics: A review of advanced materials and cooling methods. *Thermo* **5**(3), 30 (2025).

[6] Lakshminarayanan, V., & Sriraam, N. The effect of temperature on the reliability of electronic components. In *2014 IEEE International Conference on Electronics, Computing and Communication Technologies (CONECCT) 2014 Jan 6* (pp. 1–6). IEEE.

[7] Sarvar, F., Whalley, D. & Conway, P. Thermal interface materials—A review of the state of the art. *Electron. Syst. Technol. Conf.* **2**, 1292–1302 (2006).

[8] Xing, W., Xu, Y., Song, C. & Deng, T. Recent advances in thermal interface materials for thermal management of high-power electronics. *Nanomaterials* **12**(19), 3365 (2022). 36234498 10.3390/nano12193365 PMC9565324

[9] Rangarajan, S., Schiffres, S. N. & Sammakia, B. A review of recent developments in “on-chip” embedded cooling technologies for heterogeneous integrated applications. *Eng* **26**, 185–197 (2023).

[10] Chen, J., Xu, X., Zhou, J. & Li, B. Interfacial thermal resistance: Past, present, and future. *Rev. Mod. Phys.* **94**, 025002 (2022).

[11] Zhang, P. et al. A theoretical review on interfacial thermal transport at the nanoscale. *Small* **14**, 1702769 (2018). 10.1002/smll.201702769 29226601

[12] Peng, L. et al. Stable passive heat dissipation on highly thermally conductive superhydrophobic coatings based on MWCNTs and Fe2O3 doping. *J. Alloy. Compd.* **19**, 181045 (2025).

[13] Wu, Z. et al. Dielectric thermally conductive boron nitride/silica@ MWCNTs/polyvinylidene fluoride composites via a combined electrospinning and hot press method. *J. Mater. Sci. Mater. Electron.* **35**(15), 1032 (2024).

[14] Majeed, A. et al. Thermally developed radiated flow of single and multiple carbon nanotubes (SWCNTs-MWCNTs) with variable thermal conductivity. *J. Radiat. Res. Appl. Sci.* **18**(1), 101244 (2025).

[15] Tene, T. et al. Influence of MWCNT concentration on performance of Nylon/MWCNT nanocomposite-based triboelectric nanogenerators fabricated via spin coating method. *Nanoenergy Adv.* **5**(3), 9 (2025).

[16] Zeinedini, A., Akhavan-Safar, A. & da Silva, L. F. The role of agglomeration in the physical properties of CNTs/polymer nanocomposites: A literature review. *Proc. Inst. Mech. Eng. Part L: J. Mater. Des. Appl.* **2**, 14644207251316470 (2025).

[17] Wei, B. et al. Thermal interface materials: From fundamental research to applications. *SusMat* **4**(6), e239 (2024).

[18] Park, D. W. & Shim, S. E. A review on thermal conductivity of polymer composites using carbon-based fillers: Carbon nanotubes and carbon fibers. *Carbon Lett.* **11**(4), 347–356 (2010).

[19] Kim, H. S., Jang, J. U., Yu, J. & Kim, S. Y. Thermal conductivity of polymer composites based on the length of multi-walled carbon nanotubes. *Compos. B Eng.* **15**(79), 505–512 (2015).

[20] Adnan, A. W., Khan, I., Shemseldin, M. A. & Mousa, A. A. Numerical energy storage efficiency of MWCNTs-propylene glycol by inducing thermal radiations and combined convection effects in the constitutive model. *Front. Chem.* **10**, 879276 (2022). 35707459 10.3389/fchem.2022.879276 PMC9189928

[21] Alharbi, K. A. et al. Novel magneto-radiative thermal featuring in SWCNT–MWCNT/C2H6O2–H2O under hydrogen bonding. *Int. J. Modern Phys. B.* **38**(02), 2450017 (2024).

[22] Manimaran, M. et al. Critical review on the stability and thermal conductivity of water-based hybrid nanofluids for heat transfer applications. *RSC Adv.* **15**(18), 14088–14125 (2025). 40313320 10.1039/d5ra00844a PMC12044523

[23] Huang, J., Liu, X. & Du, Y. Fabrication of free-standing flexible and highly efficient carbon nanotube film/PEDOT: PSS thermoelectric composites. *J. Materiom.* **8**(6), 1213–1217 (2022).

[24] Kamalakshan, A. et al. Fabrication of flexible, adhesive, and highly conductive freestanding carbon nanotube films with poly (disulfide) main chains containing a functional polymer for high-performance carbon-based perovskite solar cells. *Sustain. Energy Fuels* **9**(16), 4352–4363 (2025).

[25] Zhang, Z., Hu, C. & Qin, Q. H. The improvement of void and interface characteristics in fused filament fabrication-based polymers and continuous carbon fiber-reinforced polymer composites: A comprehensive review. *Int. J. Adv. Manuf. Technol.* **21**, 1–41 (2025).

[26] Lee, D. K., Yoo, J., Kim, H., Kang, B. H. & Park, S. H. Electrical and thermal properties of carbon nanotube polymer composites with various aspect ratios. *Materials.* **15**(4), 1356 (2022). 35207898 10.3390/ma15041356 PMC8874980

[27] Tripathy, S. et al. Viscoelastic and thermal properties of unzipped multiwalled carbon nanotubes reinforced polyamide-6 composites. *Diam. Relat. Mater.* **1**(151), 111766 (2025).

[28] Han, Z. & Fina, A. Thermal conductivity of carbon nanotubes and their polymer nanocomposites: A review. *Prog. Polym. Sci.* **36**(7), 914–944 (2011).

[29] Anis, B. et al. Preparation, characterization, and thermal conductivity of polyvinyl-formaldehyde/MWCNTs foam: A low cost heat sink substrate. *J. Mater. Res. Technol.* **9**, 2934–2945. 10.1016/j.jmrt.2020.01.044 (2020).

[30] Yang, F., Xie, M., Yudi, Z. & Xu, X. Effect of multi-walled carbon nanotubes with different diameters on morphology and thermal and mechanical properties of flexible polyurethane foams. *Cell. Polym.* **40**(4), 165–179. 10.1177/02624893211017284 (2021).

[31] Chen, X. & Liu, Y. *Finite Element Modeling and Simulation with ANSYS Workbench* (CRC Press, New York, 2018).

[32] Kline, S. J. Describing uncertainty in single sample experiments. *Mech. Eng.* **75**, 3–8 (1953).

[33] Kabeel, A. E., Mousa, M. G. & Elsayed, M. Theoretical study of thermoelectric cooling system performance. *J. Eng. Res.* **3**(3), 10–19 (2019).

[34] Duc, H. M. et al. The effect of multiwalled carbon nanotubes on the thermal conductivity and cellular size of polyurethane foam. *Adv. Polym. Technol.* **2021**(1), 6634545 (2021).

[35] Ji, T., Feng, Y., Qin, M. & Feng, W. Thermal conducting properties of aligned carbon nanotubes and their polymer composites. *Compos. Part A Appl. Sci. Manuf.* **91**(Part 1), 351–369. 10.1016/j.compositesa.2016.10.009 (2016).

[36] Cui, Y., Li, M. & Hu, Y. Emerging interface materials for electronics thermal management: Experiments, modeling, and new opportunities. *J. Mater. Chem. C* **8**(31), 10568–10586 (2020).

[37] Moore, A. L. & Shi, L. Emerging challenges and materials for thermal management of electronics. *Mater. Today* **17**(4), 163–174 (2014).

[38] Prasher, R. Thermal interface materials: Historical perspective, status, and future directions. *Proc. IEEE* **94**(8), 1571–1586 (2006).

[39] Prasher, R. S., Shipley, J., Prstic, S., Koning, P. & Wang, J. L. Thermal resistance of particle laden polymeric thermal interface materials. *J. Heat Transfer.* **125**(6), 1170–1177 (2003).

